# Learning to Fuse Multiple Brain Functional Networks for Automated Autism Identification

**DOI:** 10.3390/biology12070971

**Published:** 2023-07-08

**Authors:** Chaojun Zhang, Yunling Ma, Lishan Qiao, Limei Zhang, Mingxia Liu

**Affiliations:** 1 The School of Computer Science and Technology, Shandong Jianzhu University, Jinan 250101, China; zhangchaojunlcu@163.com; 2 The School of Mathematics Science, Liaocheng University, Liaocheng 252000, China; myl1030@163.com (Y.M.); qiaolishan@lcu.edu.cn (L.Q.); 3 Department of Radiology and BRIC, University of North Carolina at Chapel Hill, Chapel Hill, NC 27599, USA

**Keywords:** functional connectivity network, multi-FCN fusion, adaptive weighting, autism spectrum disorder

## Abstract

**Simple Summary:**

This study aims to provide computer-aided diagnosis and valuable biomarkers for autism spectrum disorders by leveraging functional connectivity networks (FCNs) from resting-state functional magnetic resonance imaging data. We propose a novel framework for multi-FCN fusion to adaptively learn the fusion weights of component FCNs during the classifer’s learning process, guided by label information. It is simple and has better discriminability for autism spectrum disorder identification.

**Abstract:**

Functional connectivity network (FCN) has become a popular tool to identify potential biomarkers for brain dysfunction, such as autism spectrum disorder (ASD). Due to its importance, researchers have proposed many methods to estimate FCNs from resting-state functional MRI (rs-fMRI) data. However, the existing FCN estimation methods usually only capture a single relationship between brain regions of interest (ROIs), e.g., linear correlation, nonlinear correlation, or higher-order correlation, thus failing to model the complex interaction among ROIs in the brain. Additionally, such traditional methods estimate FCNs in an unsupervised way, and the estimation process is independent of the downstream tasks, which makes it difficult to guarantee the optimal performance for ASD identification. To address these issues, in this paper, we propose a multi-FCN fusion framework for rs-fMRI-based ASD classification. Specifically, for each subject, we first estimate multiple FCNs using different methods to encode rich interactions among ROIs from different perspectives. Then, we use the label information (ASD vs. healthy control (HC)) to learn a set of fusion weights for measuring the importance/discrimination of those estimated FCNs. Finally, we apply the adaptively weighted fused FCN on the ABIDE dataset to identify subjects with ASD from HCs. The proposed FCN fusion framework is straightforward to implement and can significantly improve diagnostic accuracy compared to traditional and state-of-the-art methods.

## 1. Introduction

Autism spectrum disorder (ASD) is a neurodevelopmental disease in children, mainly manifested as language communication impairment, narrow interests, and stereotyped behavior [1]. The prevalence of ASD was reported to be 0.72% in the two decades between 2000 and 2020 [2]. Although there is a growing concern about ASD, the current diagnosis of ASD is mainly based on patient behavior and simple observation of symptoms, which easily leads to a high misdiagnosis rate and, thus, delayed treatment [3]. Therefore, it is important to explore a reliable and automatic strategy to assist in the diagnosis of ASD at an early stage [4].

Resting-state functional magnetic resonance imaging (rs-fMRI), a technique that can measure the blood oxygen level-dependent (BOLD) signal of a subject at rest or in a task-free state, has been widely used in the early diagnosis of ASD [5,6]. Studies have found that ASD tends to disrupt neural connections in different brain regions of interest (ROIs) [7], and the functional connectivity network (FCN) estimated from rs-fMRI is a powerful tool to characterize subtle abnormal changes in ROIs of ASD subjects. Hence, estimating high-quality FCNs becomes a key problem in ASD identification and related biomarker discovery.

Over the past decades, many methods have been developed to estimate FCNs. Among them, the most popular is Pearson’s correlation (PC) [8]. Despite its simplicity and empirical effectiveness, PC cannot exclude the possibility of confounding influences from other brain regions, thus easily leading to misconnections among ROIs. In contrast, partial correlation can solve this problem by regressing out the effect from other ROIs. In practice, sparse representation (SR) [9] is one of an often-used scheme to calculate partial correlation, and can generally result in a more stable FCN estimation. Unlike PC and SR that only model linear correlation, Wang et al. used mutual information (MI) to measure the nonlinear relationship between ROIs [10]. A further study [11] reported that MI can confirm known functional connections and discover new ones with a clear physical explanation. Recently, Zhang et al. proposed higher-order functional connectivity (HOFC) networks that use topographical similarity information between lower-order subnetworks to quantify interactions in the brain [12]. Although the above methods have been successfully applied in many scenarios, including ASD identification, each of them only captures a single type of relationship between ROIs, making it difficult to model complex interactions in the brain.

Motivated by the concept of multiview learning, researchers try to combine information from different aspects to enhance the representation of FCN. The most direct and natural fusion method is to average differently estimated FCNs [13]. Despite its simplicity, such a traditional scheme may reduce the discriminative ability of the fused FCN, due to the fact that edge weights with positive and negative signs tend to cancel each other out. Furthermore, the average fusion strategy supposes that each FCN contributes the same weights, which makes it difficult to be consistent with the real scenarios. Unfortunately, however, there is currently no principled strategy to determine the fusion weight value of different FCNs.

In addition to the traditional average weighting method mentioned above, researchers have proposed some advanced methods to explore the potential association information between multiple FCNs. For example, Wang et al. proposed a multi-FCN fusion strategy (MVJB) that superimposes multiple FCNs into a tensor and uses the tensor decomposition to learn the joint embedding of each ROI in different views [14]. Although this method can effectively learn the commonality between multiple FCNs, it ignores the specificity among different FCNs. More seriously, this method performs fusion without utilizing supervised information and, thus, cannot adaptively adjust the fusion strategy according to the downstream task. In contrast, Gan et al. proposed a new multi-FCN fusion method (FC-kNN) based on the idea of intraclass compactness and interclass separability [15]. However, this method is limited to the fusion of two FCNs, and the fused FCNs can only contain positive edge weights, which is not suitable for most FCNs that generally have negative edge weights for modeling the inhibitory relationship between ROIs.

With the development of deep learning, researchers have used graph neural networks [16] to fuse multiple FCNs. In a recent study, Wen et al. proposed a multiview convolutional neural network (MVS-GCN) with prior brain structure to capture the potential relationships between different views (i.e., FCNs here) [17]. However, the FCNs involved in this method only contain edges estimated via linear correlation, and the multiple views are generated by simply setting different thresholds. Therefore, it is hard for such FCNs to encode the complex interaction between ROIs. Moreover, MVS-GCN is fundamentally different from traditional fusion, because it only generates a latent feature representation of different views rather than a fused FCN. In other words, we cannot achieve a specific FCN for the subject, which may result in a low model interpretability. In addition, although the GCN-based methods have shown effectiveness in brain disease classification, their models are generally complicated and contain many (hyper-) parameters. This can easily lead to the overfitting problem due to the limited training samples in the medical imaging field.

In response to the above issues, in this paper we propose an adaptive multi-FCN fusion framework for ASD classification. Specifically, for each subject, we first estimate multiple FCNs that can model different types of interactions among ROIs, including linear, nonlinear, low-order, high-order, etc. Such estimated FCNs are expected to sufficiently describe the uniqueness of the individual brain. Then, rather than choosing the traditional average fusion, we adaptively learn the fusion weights, i.e., the importance of different FCNs, under the guidance of the label information (ASD vs. healthy control (HC)) from the training samples/subjects. Moreover, we integrate the weight learning process and the downstream classification task (i.e., disease diagnosis) into a unified framework. And more notably, the obtained fusion weights are shared across all the subjects. This makes them easily generalizable to the testing samples/subjects, and can effectively reduce the risk of overfitting. The main contributions are as follows:

- An adaptive multi-FCNs fusion strategy is proposed for ASD diagnosis based on rs-fMRI by utilizing label information and diverse component FCNs, resulting in a more flexible and highly discriminative fused FCN.
- The fusion weights of component FCNs and the classifier are simultaneously optimized in a unified framework, making the model straightforward to implement and enhancing its generalization ability. This differs from the traditional FCN fusion methods which generally involve numerous hyperparameters and can easily lead to the overfitting problem on the limited medical data.
- Extensive experiments on the ABIDE datasets demonstrate the comparative performance of our method against several state-of-the-art FCNs fusion approaches.

The remainder of this paper is presented below. In Section 2, we first report the data acquisition and preprocessing procedure. Then, we propose our multi-FCN fusion method, including the mathematical model and joint optimization algorithm for ASD classification. In Section 3, we describe the experimental setup and show the experimental results. In Section 4, we discuss the effect of different parameters on classification performance and visualize discriminative features (i.e., functional connectivity). Finally, we conclude the paper in Section 5.

## 2. Materials and Methods

### 2.1. Data Acquisition and Preprocessing

In this paper, we use the raw imaging data from the Autism Brain Imaging Data Exchange (ABIDE) [18], a publicly available dataset, as the materials to test the proposed method. These data are collected from 17 international sites, and can be freely downloaded from the website http://fcon_1000.projects.nitrc.org/indi/abide/ (accessed on 1 June 2023). For training a more reliable model, we select the two largest sites in the ABIDE dataset, i.e., New York University (NYU) and University of Michigan (UM)) in this study, and report the subject demographic information in Table 1.

The NYU site includes 79 subjects with ASD and 105 HCs. Their rs-fMRI images were acquired on a clinically conventional 3.0 Tesla Allegra scanner. The imaging sequence parameters were as follows: scanning time = 6 min, TR/TE = 2000/15 ms, 33 slices, time point T = 180, flip angle = 90°, and voxel size = 3.0 × 3.0 × 4.0 mm${}^{3}$.

The UM site includes 68 subjects with ASD and 77 HCs. Their rs-fMRI images were acquired on a clinically conventional 3.0 Tesla GE Signa scanner. The imaging sequence parameters were as follows: scanning time = 10 min, TR/TE = 2000/30 ms, 40 slices, time point T = 300, flip angle = 90°, and voxel size = 3.438 × 3.438 × 3.0 mm${}^{3}$.

We preprocessed the acquired data using the Data Processing Assistant for Resting-state fMRI (DPARSF http://www.rfmri.org/DPARSF) (accessed on 1 June 2023) [19] based on a recognized pipeline: (1) Removal of the first 5 rs-fMRI volumes for each subject; (2) slice-timing correction and head motion correction; (3) registration in Montreal Neurological Institute (MNI) space [20] with a resolution of $3\times 3\times 3\;{\mathrm{mm}}^{3}$; (4) regression of interfering signals (ventricle, white matter, global signals, and head motion described by Friston 24-parameters model); (5) a temporal high-pass filter of 0.01–0.1 Hz is used to reduce the effects of heartbeat and breathing. Finally, the brain is divided into 116 ROIs based on the Automatic Anatomical Labeling (AAL) atlas [21], and a representative BOLD signal is extracted from each ROI by an averaging strategy [22] for the subsequent FCN construction.

### 2.2. Proposed Method

**Figure 1:**
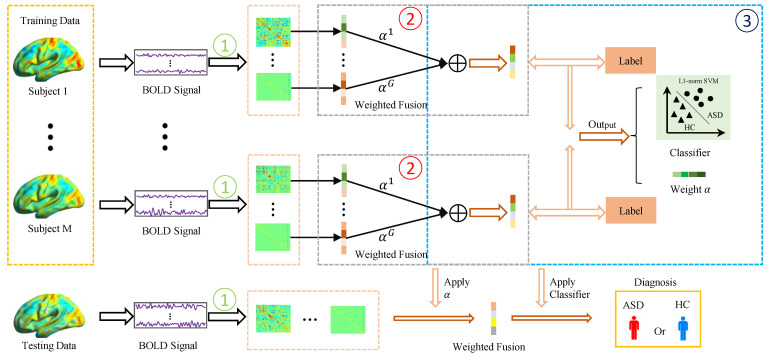
The proposed multi-functional connectivity networ (FCN) fusion framework. (1) Multiple initial brain function networks are estimated by traditional methods for each subject; (2) fuse these FCNs with the weight combination; (3) incorporate the weight learning into the L1-norm support vector machine (SVM) classifier, with the result that simultaneously optimizes the weights $\alpha^{j}\left(j=1,\cdots ,G\right)$ and the classifier. Finally, the model parameters in (3) are applied for autism spectrum disorder (ASD) diagnosis.

As mentioned earlier, the traditional FCN estimation methods only can capture a single type of relationship between ROIs, which makes it difficult to model intricate connections in the brain. To obtain more reliable brain connections, it has become increasingly popular to fuse multiple FCNs estimated from different views or based different methods. The simplest and most straightforward fusion method is to average these FCNs directly. However, such an averaging strategy easily suffers from the following issues. First, it assigns the same weight/importance for each FCN, which is not flexible enough to encode the complicated brain correlations in the real scenarios. Second, the traditional averaging fusion method is unsupervised and cannot adaptively adjust the fusion strategy according to the downstream tasks. Thus, the fused FCN is not necessarily beneficial to the ASD classification performance. To solve these problems, we develop a novel multi-FCN fusion method, in which the fusion weights are learned automatically from the data with the guidance of the label information. By combining the learning of fusion weights with the classification task, the resultant FCNs may have stronger discriminative ability. The pipeline of the proposed muti-FCN fusion framework is shown in Figure 1.

#### 2.2.1. Joint Multi-FCN Fusion and Disease Classification

In our multi-FCN fusion framework, in order to model the complex interactions between ROIs, we first estimate a set of FCNs for each subject based on different methods, denoted as $W^{j}\left(j=1,\cdots ,G\right)$, where *G* is the number of involved methods that will be introduced shortly in Section 3.1. Then, we fuse these FCNs through a set of weights $\alpha^{j}\left(j=1,\cdots ,G\right)$, each of which corresponds to an FCN. In other words, the fused FCN $W=\sum_{i=1}^{G}\alpha^{j}W^{j}$. Finally, we integrate the fused FCN with fusion weights and label information into a unified framework based on the L1-norm support vector machine (L1-norm SVM) [23], with the aim to obtain the fusion weights that are conducive to minimizing classification errors. As a result, we have the joint multi-FCN fusion and disease classification model as follows:

(1) $$\begin{array}{cc} \min_{C,\alpha }\; & {∥C∥}_{1}+\lambda_{1}\sum_{i=1}^{M}{\left(max\left(0,1-y_{i}C^{T}\sum_{j=1}^{G}\alpha^{j}W_{i}^{j}\right)\right)}^{2}+\lambda_{2}{∥\alpha ∥}^{2}\\ s.t.\; & \alpha^{j}\geq 0,\sum_{j=1}^{G}\alpha^{j}=1 \end{array}$$

where $W_{i}^{j}\left(i=1,\cdots ,M,j=1,\cdots ,G\right)$ represents the adjacency matrix (without loss of generality, the $W_{i}^{j}$ has been concatenated into a vector) of FCN corresponding to the *i*-th subject, estimated by the *j*-th methods, and $y_{i}\in \left\{-1,1\right\}$ is the label of the *i*-th subject. In other words, y takes the value -1 or 1, where -1 indicates HC and 1 indicates a patient with ASD. *C* is the parameter vector in the L1-norm SVM [23]. The L1-norm ${∥\cdot ∥}_{1}$ aims to make the parameter vector *C* sparse, which in fact embeds a feature selection operation in the SVM classifier. As a result, we do not need an extra feature selection step for the fused FCNs. Additionally, to prevent the model from degenerating to the trivial fusion weight value, i.e., only one 1 and G−1 zeros in $\alpha =(\alpha^{1},\cdots ,\alpha^{G})$, we include an L2-norm regularization term ${∥\alpha ∥}^{2}$ in the classification model. The $\lambda_{1}$ and $\lambda_{2}$ are two regularization parameters to control the balance of three terms in the objective function. At the same time, our method constrains $\alpha^{j}\geq 0,\sum_{j=1}^{G}\alpha^{j}=1$, which not only avoids the trivial solution (i.e., $\alpha^{j}=0$, ∀j), but also gives the weight values $\alpha^{j}$ a clear probabilistic interpretation.

In contrast to the traditional averaging strategy in which α=(1/G,⋯,1/G), our model can adaptively learn a set of weights for the involved FCNs. Therefore, the model proposed in this paper is more flexible than the traditional schemes. In addition, the fusion weights and downstream classifications are integrated into a unified framework, which may improve the discriminative power of the final fused FCN under the guidance of label information.

#### 2.2.2. Optimization Algorithm

Note that two variables, *C* and α, are involved in Equation (1). Here, we use the alternating optimization (AO) algorithm to solve the problem.

Step 1: When α is fixed, we update *C*, and the model can be simplified to the following L1-norm SVM [23]:

(2) $$\begin{array}{cc} \min_{C}\; & {∥C∥}_{1}+\lambda_{1}\sum_{i=1}^{M}{\left(max\left(0,1-y_{i}C^{T}\sum_{j=1}^{G}\alpha^{j}W_{i}^{j}\right)\right)}^{2} \end{array}$$

Without loss of generality, we express $\sum_{j=1}^{G}\alpha^{j}W_{i}^{j}=W_{i}$, that is, Equation (2) can be rewritten as

(3) $$\begin{array}{cc} \min_{C}\; & {∥C∥}_{1}+\lambda_{1}\sum_{i=1}^{M}{\left(max\left(0,1-y_{i}C^{T}W_{i}\right)\right)}^{2} \end{array}$$

Equation (3) is a classical L1-SVM model. At present, researchers have developed many algorithms to solve it [23,24,25]. In this paper, we use the toolbox LIBLINEAR to achieve the optimal solution of *C* [23].

Step 2: With fixed *C*, we then update α, and the model can be simplified as

(4) $$\begin{array}{cc} \min_{\alpha }\; & \lambda_{1}\sum_{i=1}^{M}{\left(max\left(0,1-y_{i}C^{T}\sum_{j=1}^{G}\alpha^{j}W_{i}^{j}\right)\right)}^{2}+\lambda_{2}{∥\alpha ∥}^{2}\\ s.t.\; & \alpha^{j}\geq 0,\sum_{j=1}^{G}\alpha^{j}=1 \end{array}$$

Since $max(0,1-y_{i}C^{T}\sum_{j=1}^{G}\alpha^{j}W_{i}^{j})$ in Equation (4) is nonconvex with respect to α, we introduce a slack variable $\xi_{i}\geq 0$, and Equation (4) can be rewritten as

(5) $$\begin{array}{cc} \min_{\alpha }\; & \lambda_{1}\sum_{i=1}^{M}\xi_{i}^{2}+\lambda_{2}{∥\alpha ∥}^{2}\\ s.t.\; & \xi_{i}\geq 1-y_{i}C^{T}\sum_{j=1}^{G}\alpha^{j}W_{i}^{j},\xi_{i}\geq 0,\alpha^{j}\geq 0,\sum_{j=1}^{G}\alpha^{j}=1 \end{array}$$

Note that Equation (5) is a quadratic programming problem; thus, we use the ready-made quadprog function [26] in MATLAB toolbox to solve it. Finally, we summarize the optimization algorithm for solving Equation (1) in Algorithm 1. The description above represents the training process in Figure 1. For a new subject, we used the testing process in Figure 1, taking multiple FCNs of the new subject, applying the weights α learned from the training set for weighted fusion, and then using the learned classifier to make class judgments.
**Algorithm 1** Algorithm of the proposed model.**Input:** Input data $W=W_{i}^{j}\left(i=1,\cdots ,M;j=1,\cdots ,G\right)$, *y*; parameter $\lambda_{1},\lambda_{2}$; maximum iteration number: MaxIter; and the iteration stopping threshold ϵ. **Output:** Weight vector *C*, fusion weight vector α. **Initialize:**$C\in R^{M}$, choose α=(1/G,⋯,1/G). **For**
i=1,2,⋯,MaxIter   Step 1: Fixed α, updated *C*      By solving Equation (3)   Step 2: Fixed *C*, updated α      By solving Equation (5)   Calculate Equation (1) as $F_{i}$. If $|F_{i+1}-F_{i}|<\epsilon$   Break and Return C,α. End **End**


## 3. Experiments

In the experimental part, we first introduce the construction of multiple FCNs from different perspectives, then describe the comparison methods, and finally give the experimental settings and results.

### 3.1. Estimated Multi-FCN

As mentioned earlier, our proposed fusion scheme is not limited by the number of FCNs. To encode the diverse and complex interactions between ROIs in the brain, we use four popular methods, including PC [8], SR [9], MI [10], and HOFC [12], to construct multiple FCNs prior to the fusion representation. These methods are able to model the relationship between ROIs from different perspectives. In particular, PC measures full correlation, SR captures partial correlation, MI models nonlinear relationships, and HOFC encodes higher-order relationships. In Table 2, we list the models/formulas used to estimate FCNs in different methods.

PC measures the full correlations between two ROIs based on Equation (6) in Table 2, where $x_{i},x_{j}\in R^{T}(i,j=1,\cdots ,116$) are the BOLD signals of the *i*-th and *j*-th ROI, respectively. ${\bar{x}}_{i}$ (or ${\bar{x}}_{j}$) is the mean of $x_{i}$ (or $x_{j}$).

SR captures the partial correlations between different ROIs. The first term of Equation (7) in Table 2 is the data fitting term used to model the part of correlations between different ROIs, and the second term is the L1-regularizer used to encode the sparse prior of FCN. λ is a regularization parameter to control the balance of two terms in the objective function. It is worth noting that the regularization parameter of SR is fixed to be $\lambda =2^{-5}$ for matching the brain network density in other methods, which also corresponds to the settings in the previous research [27]. In addition, the FCN estimated by SR is asymmetric; thus, we simply symmetrize it by $S=(S+S^{T})/2$ in our experiment [27].

MI measures the nonlinear relationship between two ROIs based on Equation (8), as shown in Table 2, where *p*(*x*${}_{i}$,*x*${}_{j}$), $p(x_{i})$ and $p(x_{j})$ are the joint probability distribution and marginal probability distribution of $x_{i}$ and $x_{j}$, respectively.

HOFC measures the high-order interaction information between two ROIs, implemented on top of the PC-estimated FCN. Specifically, we first construct a fully connected low-order FCN based on PC. Then, the edge weights of such low-order FCN are considered as new feature representations to estimate a higher-order FCN by conducting PC operation again [12]. As shown in Equation (9), $P_{i}$ represents the *i*-th column of the low-order FCN adjacency matrix estimated by the first PC operation.

### 3.2. Methods for Comparison

In our experiments, we compare eight methods, including four single methods (i.e., PC [8], SR [9], MI [10], and HOFC [12]) shown in Table 2, and four fusion methods (i.e., the simple averaging weighted method, MVJB [14], FC-kNN [15], and MVS-GCN [17]). In the following, we give a high-level description of the four FCN fusion methods.

- Average: It fuses multiple FCNs by distributing the same weight for each FCN.
- MVJB: This method superimposes multiple FCNs into a tensor and uses tensor decomposition to learn a joint embedding representation of each ROI. Then PC is used to calculate the correlation between the embedding representations of ROIs to obtain the fused FCN [14].
- FC-kNN: It uses the criterion of intraclass compactness and interclass separability to fuse the commonality and specificity of two FCNs [15].
- MVS-GCN: MVS-GCN first generates dense FCNs and then binarizes them into multiple FCNs by different thresholds. Rather than fusing these FCNs directly, it uses multitask embedding learning to extract potential correlation features from different FCNs [17].

### 3.3. Experimental Setting

As mentioned previously, we choose four representative methods (i.e., PC, SR, MI, and HOFC) to construct multiple FCNs for each subject and normalize their adjacency matrices to have entries in the interval of [−1,1]. Since the FCNs constructed by all methods are symmetric, we only consider the upper triangular elements as their representation; thus, each FCN has 6670 features. Then, the features corresponding to multiple FCNs are placed into the proposed fusion framework, which is guided by label information for adaptive weight learning and used in the classification task of ASD.

**Figure 2:**
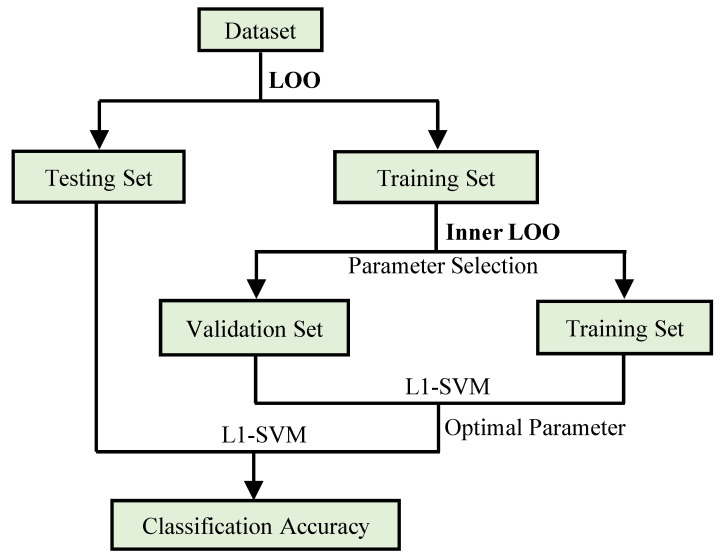
Selection of the optimal parameter pipeline via leave-one-out cross-validation.

Due to the limited number of subjects, we test the proposed method using leave-one-out (LOO) cross-validation. That is, one subject is kept for testing and the remaining subjects are used for training the model. This process is repeated until each subject is tested once. For the regularization parameter $\lambda_{2}$, we search its optimal value in the range of [0,1,⋯,5,6] by an inner LOO process based on an independent validation set, as shown in Figure 2. For the parameter $\lambda_{1}$, we use the default value of 1 as in the L1-norm SVM [23].

In our experiments, we use several popular indicators [28,29,30], including accuracy ($ACC=\frac{TP+TN}{TP+FP+TN+FN}$), sensitivity ($SEN=\frac{TP}{TP+FN}$), specificity ($SPE=\frac{TN}{TN+FP}$), balance accuracy ($BAC=\frac{SEN+SPE}{2}$), positive predictive value ($PPV=\frac{TP}{TP+FP}$), negative predictive value ($NPV=\frac{TN}{TN+FN}$), F1 score ($F1=\frac{2\times TP}{2\times TP+FP+FN}$), and area under the ROC curve (AUC) to assess the classification performance of the involved methods, where TP, TN, FP, and FN are true positive, true negative, false positive, and false negative, respectively.

### 3.4. Classification Performance

The classification results for NYU and UM sites are shown in Table 3 and Table 4, respectively. According to the classification results, we can observe that the proposed fusion framework achieves better results than the comparison methods. In particular:

- Most of the muti-FCN fusion methods generally achieve better recognition performance than single FCN method. This further illustrates that it is not easy to acquire a good representation of brain only using a single type of FCN since the interaction between different ROIs in the real brain is extremely complex.
- The simple average-weighted approach cannot work well on the used two datasets. In contrast, our proposed method improves the ASD classification performance by 6.52% and 11.03% at NYU and UM sites, respectively. This may benefit from the adaptively optimized fusion weights combined with the label information for each type of FCN, as shown in Equation (1).
- Compared to MVJB [14] and FC-kNN [15], our method also contributes significantly to the improvement in accuracy. On the one hand, this is due to the fact that we incorporate the fused weight learning into the classification task, which may help to improve the discriminative ability of the final fused FCN. On the other hand, our method is not limited by the number of fused FCNs, and thus can obtain information from more FCNs.
- Despite its simplicity, our method can outperform MVS-GCN [17], a deep-learning-based multiview learning scheme. The possible reason is that the MVS-GCN framework needs to determine a lot of hyperparameters, which easily incurs the difficulty in parameter selection and may cause the overfitting problem, since the amount of training data is limited in our experiments.

We further performed a statistical analysis to assess the significance of differences between the probability scores obtained from the proposed method and the eight comparative methods. As shown in Table 5, the *p*-values obtained from the t-tests are below the significance level (p<0.05), which indicates that the proposed method is significantly different from each of the comparison methods.

## 4. Discussion

### 4.1. Hyperparameter Analysis

**Figure 3:**
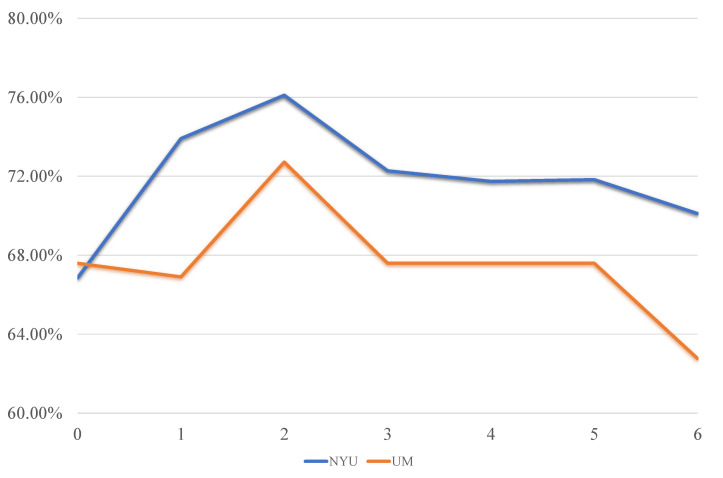
The effect of the regularization parameter $\lambda_{2}$ on the classification performance.

In general, the values of the free parameters involved in the model may influence the final classification performance. In this section, we only focus on the regularization parameter $\lambda_{2}$ in the proposed model, since the parameter $\lambda_{1}$ is fixed to the default value as in L1-norm SVM [23]. In Figure 3, we show its impact on the classification performance for the NYU and UM sites, respectively.

It can be found that the performance of the proposed model is sensitive to the value of the parameter $\lambda_{2}$, showing an overall trend of first increasing and then decreasing. In particular, when $\lambda_{2}=0$, the classification accuracy is low, due to that in this case the proposed model degenerates into the L1-norm SVM, leading to a trivial fusion weight value, that is, one 1 and three 0 in $\alpha^{j}\left(j=1,\cdots ,4\right)$. As a result, only one single FCN, but not the multi-FCN fusion, is used as the feature to classify ASD. The performance gradually increases till the value of $\lambda_{2}$ reaches the best at $\lambda_{2}=2$. Then, when $\lambda_{2}$ is too big, the final fusion weights tend to average out, that is, the α closes to (1/4,1/4,1/4,1/4). This limits the flexibility of fusion and thus leads to a decrease in classification performance.

### 4.2. Classification Performance with Different Numbers of Fused FCNs

**Figure 4:**
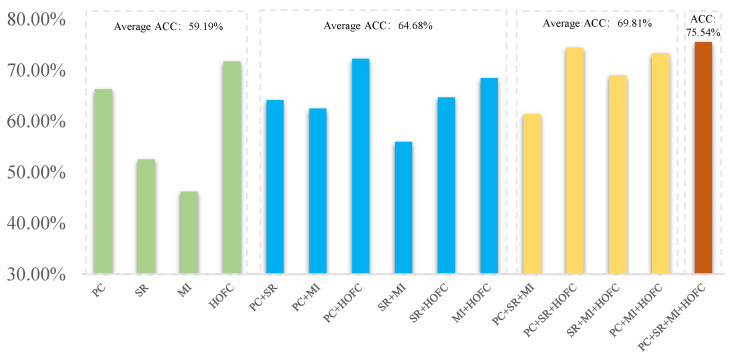
Classification performance at NYU site with different numbers of fused FCNs. Single, two, three, and four FCNs are represented in green, blue, yellow, and orange, respectively.

Further, under the proposed multi-FCN fusion framework, we discuss the effect of different numbers of FCNs on the ASD classification performance, as shown in Figure 4. In this experiment, the regularized parameters are set to be the same in order to make a fair comparison. It can be seen, from the average accuracy of Figure 4, that either fusing two FCNs, three FCNs, or four FCNs results in a better ASD classification performance compared to the four single FCN methods, and the overall trend is increasing. This verifies that multi-FCN fusion is helpful for the improvement of classification performance. At the same time, the experiment demonstrates the flexibility of our proposed fusion framework, that is, any existing FCN estimation method can be merged into our model and is not limited by the number of FCNs. This is crucial for capturing complex correlations between ROIs in unknown real brain networks [31,32].

### 4.3. Discriminative Features

**Figure 5:**
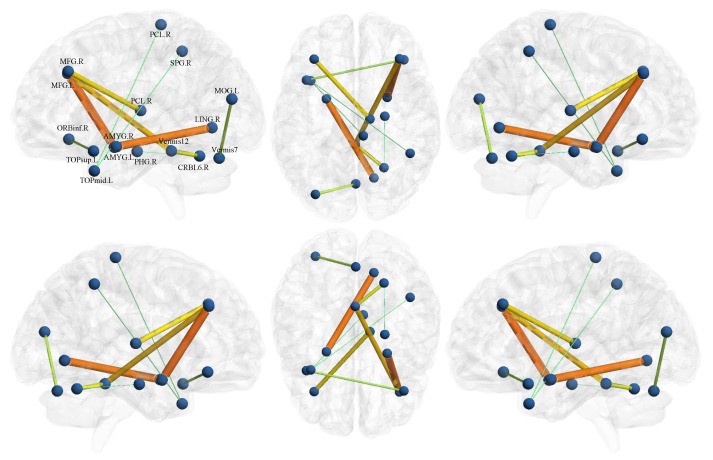
The top ten most discriminating features selected for ASD classification at the NYU site. The thickness of the solid line connecting two brain regions of interests (ROIs) indicates the strength of the connection.

As mentioned earlier, we learn fusion weights and classifiers by alternating optimization in a unified framework. It is worth noting that in such a process of optimization, the feature selection is also finished since the classification vector *C* in Equation (1) uses the L1-norm. To explore the disease-related brain region and connectivity, we visualize the top ten most discriminating features using the BrainNet Viewer toolbox https://www.nitrc.org/projects/bnv/ (accessed on 1 June 2023) [33], as shown in Figure 5. From it, we find that the most discriminating functional connections exist between the middle frontal gyrus, amygdala, and lingual gyrus. Researches report that this connection generally has a significant impact on the cognitive, behavioral, and emotional impairment in patients with ASD [34]. In addition, the ROIs associated with the top discriminant features also include orbital inferior frontal gyrus, parahippocampal gyrus, and paracentral lobules, most of which are consistent with previous works on ASD classification task [35,36,37].

The identified discriminative ROIs hold potential as biomarkers for ASD, playing a crucial role in assisting diagnosis. By analyzing the activity patterns of these specific ROIs in patients with ASD compared to healthy individuals, healthcare professionals can obtain a reliable basis for the diagnosis of ASD. This advancement opens up possibilities for early screening and intervention in ASD, leading to improved patient outcomes. The incorporation of these biomarkers into current ASD diagnosis procedures could enhance the accuracy and efficiency of diagnosis.

### 4.4. Fusion Weight Analysis

Our proposed method is to adaptively learn the fusion weights of different FCNs combined with the downstream classification task, aiming to make the final fused FCN have a higher discriminability. Here, we analyze the weights learned from the NYU site based on the experiments in Section 3. Specifically, we average the α’s learned in all LOO loops and obtain the final fusion weight vector α=(0.168,0.317,0.000,0.515) corresponding to PC, SR, MI, and HOFC, respectively. We note that HOFC, the FCN with the best performance among the four single methods, as shown in Table 3, has the greatest contribution (i.e., the biggest weight) to the final classification performance, and accordingly, the FCN with poor performance, such as MI, has a smaller contribution. Especially for MI, the corresponding weight tends to be zero. This indicates that the learned fusion of FCNs has the potential discriminative ability, possibly benefiting from the combination with the classification task. In addition, the number of approximate zero in the fusion weight vector α heavily relies on the values of regularization parameter $\lambda_{2}$ which, in essence, balances the sparsity and evenness of α, as mentioned in the proposed model. This further shows that our proposed fusion framework can not only improve the discrimination of the resulting FCN, but also has a strong flexibility and interpretability.

### 4.5. Time Complexity Analysis

In our model, the component FCNs are pregenerated, so we ignore this part of the time complexity calculation. In our proposed Equation (1), the fusion of component FCNs requires AO to solve *C* and α. Their time complexity is O(M) and O(4M), respectively, where *M* denotes the number of subjects and 4M denotes the number of weights to optimize for each subject. The time complexity of MVJB, FC-kNN, and MVS-GCN are O(MKl), $O(lMn^{2})$, and $O(lM^{2}n)$, respectively, where *l*, *K*, and *n* are the number of iterations, views, and ROIs.

In Table 6, we give the training times for the proposed model and the compared state-of-the-art methods on the NYU site. As observed in the table, our method demonstrates the least amount of training time, which can be attributed to the simultaneous feature selection and classification tasks enabled by the L1-norm SVM used in our model.

### 4.6. Comparison with State-of-the-Art Methods

To provide a comprehensive evaluation of our proposed method, we compared its performance with three state-of-the-art FCNs fusion methods on ASD classification tasks. Specifically, Kang et al. proposed a deep-learning-based multiview ensemble learning (MEL) network that used stacked denoising self-encoders to obtain multi-FCNs [38]. Kam et al. proposed a discriminative restricted Boltzmann machine (DRBM) which utilized hierarchical clustering to construct multi-FCNs to identify discriminative ROIs [39]. Niu et al. proposed a multichannel deep attention neural network (multichannel DANN) that utilized complementary information from different brain atlases for ASD diagnosis [40].

In Table 7, we present the results obtained from these comparison methods. It is worth noting that we directly compare our classification performance with the reported results in the original articles of three comparative methods. As can be seen, our method achieves better performance on metrics ACC and AUC, which further confirms the effectiveness of the proposed method in accurately identifying ASD. One possible reason is that the fused FCNs constructed by our method combines different types of correlations among brain regions, including full correlations, partial correlations, nonlinear relationship, and high-order correlations, which makes it more possible to model the complex brain. In contrast, the fused FCNs by three comparative methods are all based on single full correlations estimated by PC, although they use different fusion strategies. This may be insufficient to capture the complex interaction between brain regions. The second reason is that the three comparative fusion methods all employ deep neural network architectures, which are more prone to overfitting when dealing with limited amounts of medical images datasets.

### 4.7. Strengths and Limitations

In this subsection, we discuss the strengths and limitations of the proposed method to provide a comprehensive evaluation.

We identify several strengths. Firstly, our method addresses the challenge of learning fusion weights for FCNs, which is crucial to model the complex brain. By learning the fusion weights adaptively from the data combined with the downstream classification task, our approach enhances the discriminability of the final fused FCN for ASD diagnosis. Secondly, our fusion method is simple, straightforward, and easy to interpret compared to other recent multi-FCN fusion works, such as Gan [15] and Wen et al. [17]. These existing methods are complex and require many (hyper-) parameters to learn, which is generally an uneasy task based on limited training data. Lastly, our proposed fusion framework is very flexible. It is not limited by the number of FCNs, and does not require the FCNs to have positive edge weights. This means that any FCNs, including those achieved through the FCN fusion methods mentioned above, can be seamlessly integrated into our model.

However, our model also has some limitations. One is that it is based on a strong assumption that all subjects share the same fusion weights of FCNs, which ignores the heterogeneity across the subjects to some extent. In addition, the current fusion strategy only is based on a single modular data, i.e., rs-fMRI, and does not incorporate information from other modular data. These limitations will be addressed in our future work.

## 5. Conclusions

In this paper, we proposed a simple multi-FCN fusion strategy in which the fusion weights are optimized under the guidance of label information for ASD identification. In particular, we incorporated the fusion of different types of FCNs into the classifier based on L1-norm SVM, with the aim to simultaneously learn the fusion weight (i.e., the importance of different FCNs) and the classifier, while feature (i.e., brain connectivity) selection is also incorporated into such a unified framework. This makes the model simple but obtains the final fused FCN with good discriminability. We validated the effectiveness of the proposed method to identify ASD patients on the ABIDE dataset, achieving 75.54% and 71.72% accuracy on the NYU and UM sites, respectively. In the future work, we plan to further integrate the variability between subjects by learning different weights for each subject separately. Specifically, we plan to optimize different fusion weights for each subject by introducing a set of hidden variables with the aim to further improve its performance [41]. Additionally, we currently focus on the fusion of FCNs based on a single modular data, i.e., rs-fMRI. To make full use of the complementary information of different modular data, we plan to explore the fusion of FCNs from different modular data, including structural MRI and positron emission tomography (PET) data. This multimodal fusion has the potential to provide a more comprehensive and robust representation of human brain.

## Figures and Tables

**Table 1:** Subject demographics from New York University (NYU) and University of Michigan (UM) sites in the Autism Brain Imaging Data Exchange (ABIDE) dataset. FIQ, full-scale intelligence quotient.

Datasets	Class	Gender (M/F)	Age (Years)	FIQ
NYU	ASD (N = 79)	68/11	14.52±6.97	107.92±3.15
	HC (N = 105)	79/26	15.81±6.25	113.15±2.45
UM	ASD (N = 68)	58/10	13.13±2.41	105.46±17.28
	HC (N = 77)	59/18	14.79±3.57	108.12±9.80

**Table 2:** Several popular FCN estimation methods.

Method	Definition
Pearson’s correlation (PC)	(6) $P_{ij}=\frac{{\left(x_{i}-{\bar{x}}_{i}\right)}^{T}\left(x_{j}-{\bar{x}}_{j}\right)}{\sqrt{{\left(x_{i}-{\bar{x}}_{i}\right)}^{T}\left(x_{i}-{\bar{x}}_{i}\right)}\sqrt{{\left(x_{j}-{\bar{x}}_{j}\right)}^{T}\left(x_{j}-{\bar{x}}_{j}\right)}}$
Sparse representation (SR)	(7) $S_{ij}=arg\min_{S_{ij}}\sum_{i=1}^{M}(∥x_{i}-\sum_{j\neq i}S_{ij}x_{j}{∥}^{2}+\lambda \sum_{j\neq i}|S_{ij}\left|\right)$
Mutual Information (MI)	(8) $M_{ij}=\sum_{i}\sum_{j}p\left(x_{i},x_{j}\right)\log\frac{p(x_{i},x_{j})}{p\left(x_{i}\right)p\left(x_{j}\right)}$
Higher Order Functional Connections (HOFC)	(9) $H_{ij}=\frac{{\left(P_{i}-{\bar{P}}_{i}\right)}^{T}\left(P_{j}-{\bar{P}}_{j}\right)}{\sqrt{{\left(P_{i}-{\bar{P}}_{i}\right)}^{T}\left(P_{i}-{\bar{P}}_{i}\right)}\sqrt{{\left(P_{j}-{\bar{P}}_{j}\right)}^{T}\left(P_{j}-{\bar{P}}_{j}\right)}}$

**Table 3:** Performance comparison of NYU site. Best results are denoted by bold values.

Datasets	Method	ACC	SEN	SPE	BAC	PPV	NPV	F1	AUC
NYU	PC	66.30%	58.23%	72.38%	65.30%	61.33%	69.72%	59.74%	70.56%
	SR	62.50%	43.03%	77.14%	60.09%	58.62%	64.29%	49.64%	66.74%
	MI	46.20%	40.51%	50.48%	45.49%	38.10%	53.00%	39.26%	41.29%
	HOFC	71.74%	62.03%	79.05%	70.54%	69.01%	73.45%	65.33%	77.20%
	Average	69.02%	59.49%	76.19%	67.84%	65.28%	71.43%	62.25%	73.66%
	MVJB	74.46%	64.56%	81.90%	73.23%	72.86%	75.44%	68.46%	78.72%
	FC-kNN	70.65%	64.56%	75.24%	69.90%	66.23%	73.83%	65.38%	75.86%
	MVS-GCN	72.28%	63.29%	79.05%	71.17%	69.44%	74.11%	66.23%	77.53%
	Proposed	**75.54**%	**65.82**%	**82.86**%	**74.34**%	**74.29**%	**76.32**%	**69.80**%	**79.07**%

**Table 4:** Performance comparison of UM site. Best results are denoted by bold values.

Datasets	Method	ACC	SEN	SPE	BAC	PPV	NPV	F1	AUC
UM	PC	56.55%	55.88%	57.14%	56.51%	53.52%	59.46%	54.68%	62.18%
	SR	51.72%	50.00%	53.25%	51.62%	48.57%	54.67%	49.28%	52.06%
	MI	49.66%	22.06%	**74.03**%	48.04%	42.86%	51.82%	29.13%	41.75%
	HOFC	62.07%	55.88%	67.53%	61.71%	60.32%	63.41%	58.02%	66.12%
	Average	60.69%	51.47%	68.83%	60.15%	59.32%	61.13%	55.12%	65.09%
	MVJB	63.45%	63.24%	63.64%	63.44%	60.56%	66.22%	61.87%	65.16%
	FC-kNN	65.52%	63.24%	67.53%	65.38%	63.24%	67.53%	63.25%	66.14%
	MVS-GCN	68.27%	70.59%	66.23%	68.41%	64.86%	71.83%	67.60%	71.56%
	Proposed	**71.72**%	**70.59**%	72.73%	**71.66**%	**69.57**%	**73.68**%	**70.07**%	**77.35**%

**Table 5:** Results of the statistical significance analysis between the proposed method and the eight comparison methods.

Pairwise Comparison	*p*-Value	p<0.05
Proposed vs. PC	$3.65\times 10^{-2}$	Yes
Proposed vs. SR	$2.64\times 10^{-2}$	Yes
Proposed vs. MI	$2.55\times 10^{-2}$	Yes
Proposed vs. HOFC	$1.28\times 10^{-2}$	Yes
Proposed vs. Average	$2.94\times 10^{-2}$	Yes
Proposed vs. MVJB	$1.46\times 10^{-2}$	Yes
Proposed vs. FC-kNN	$1.96\times 10^{-2}$	Yes
Proposed vs. MVS-GCN	$1.65\times 10^{-3}$	Yes

**Table 6:** The training time (in seconds) of the proposed model and the compared methods on the NYU site.

Methods	Proposed	MVJB	FC-kNN	MVS-GCN
Time	1480 s	1832 s	2125 s	2658 s

**Table 7:** Comparison of the classification performance of the proposed method with state-of-the-art methods on the NYU site, and the best results are shown in bold.

Method	ACC	SEN	SPE	BAC	PPV	NPV	F1	AUC
MEL	74.60%	-	-	-	-	-	72.20%	74.30%
DRBM	75.24%	61.33%	**85.71**%	-	**82.10**%	-	-	73.73%
Multichannel DANN	70.91%	**72.02**%	69.92%	-	75.82%	-	**73.83**%	-
Proposed	**75.54**%	65.82%	82.86%	**74.34**%	74.29%	**76.32**%	69.80%	**79.07**%

## Data Availability

ABIDE is a publicly available dataset that can be downloaded for free on the website of http://fcon_1000.projects.nitrc.org/indi/abide/ (accessed on 11 March 2023).

## Abbreviations

The following abbreviations are used in this manuscript:

FCN	Functional connectivity network
ASD	Autism spectrum disorder
ROI	Regions of interest
HC	Healthy control
ABIDE	Autism Brain Imaging Data Exchange
BOLD	Blood oxygen level-dependent
rs-fMRI	Resting-state functional magnetic resonance imaging
PC	Pearson’s correlation
SR	Sparse representation
MI	Mutual information
HOFC	Higher-order functional connectivity
NYU	New York University
UM	University of Michigan
AO	Alternating optimization
SVM	Support vector machine
LOO	Leave-one-out
